# Outcomes and Risk Factors Associated with *Clostridium difficile* Diarrhea in Hospitalized Adult Patients

**DOI:** 10.1155/2015/346341

**Published:** 2015-05-25

**Authors:** Daniela Zilio Larentis, Regis Goulart Rosa, Rodrigo Pires dos Santos, Luciano Zubaran Goldani

**Affiliations:** ^1^Section of Infectious Diseases, Hospital de Clínicas de Porto Alegre, Universidade Federal do Rio Grande do Sul, 90035-903 Porto Alegre, RS, Brazil; ^2^Hospital Infection Control Committee, Hospital de Clínicas de Porto Alegre, Universidade Federal do Rio Grande do Sul, 90035-903 Porto Alegre, RS, Brazil

## Abstract

*Background*. The epidemiology of *Clostridium difficile* infection has changed over time. Therefore, it is essential to monitor the characteristics of patients at risk of infection and factors associated with poor prognosis. *Objective*. To evaluate factors associated with *C. difficile* infection and with poor prognosis in those with documented *C. difficile* colitis. *Methods*. A retrospective case-control study of 75 patients with documented *C. difficile* colitis and 75 controls with hospital-acquired diarrhea of other causes. Stepwise multiple logistic regression was used to identify factors associated with *C. difficile* infection among patients with hospital-acquired diarrhea. *Results*. Previous antibiotic treatment (odds ratio (OR), 13.3; 95% confidence interval (CI), 1.40–126.90), abdominal distension (OR, 3.85; 95% CI, 1.35–10.98), and fecal leukocytes (OR, 8.79; 95% CI, 1.41–54.61) are considered as predictors of *C. difficile* colitis; anorexia was negatively associated with *C. difficile* infection (OR, 0.15; 95% CI, 0.03–0.66). Enteral tube feeding was independently associated with a composite outcome that included in-hospital mortality, intensive care unit admission, and treatment failure (OR, 3.75; 95%CI, 1.24–11.29). *Conclusions*. Previous antibiotic use and presence of fecal leukocytes in patients with hospital-acquired diarrhea are associated with *C. difficile* colitis and enteral tube support with complications associated with *C. difficile* colitis.

## 1. Introduction

*Clostridium difficile* is a Gram-positive, strictly anaerobic, spore-forming bacterium.* Clostridium difficile* infection (CDI) is an important hospital-acquired condition associated with the use of antibiotics [1, 2]. CDI has been of concern over the last decade because of its significant morbidity and mortality and the high associated healthcare costs. Some patients remain asymptomatic after exposure, whereas in others the manifestations vary from mild diarrhea to fulminant colitis. Colonization of the intestinal tract occurs via the fecal-oral route in patients with disruption of normal intestinal flora, especially that related to the use of antibiotics in immunosuppressed patients or to the use of proton pump inhibitors [3–5]. Pathogenic strains of* C. difficile* produce potent toxins (A and B), which are responsible for the clinical manifestations in humans [6]. Over the past decade, CDI has increased in both frequency and severity in the USA and in other countries [7–9]. CDI has reportedly been widely identified in prospective laboratory surveillance and outbreaks [10–13]; however, there are few reports on the frequency and impact of CDI in different institutions, especially in Latin America. Accordingly, we performed the present study to determine factors associated with* C. difficile* infection among patients with hospital-acquired diarrhea and with poor prognosis among those with documented* C. difficile* colitis in a tertiary care Brazilian hospital.

## 2. Materials and Methods

### 2.1. Study Design, Patients, and Definitions

This retrospective case-control study was conducted at the Hospital de Clínicas de Porto Alegre, an urban tertiary referral academic center (700 beds) in Porto Alegre, Brazil. In this study, all samples of stool in the hospital's microbiology laboratory electronic database between January 2010 and July 2012 were identified. These samples were analyzed for the presence of toxins A and B of* C. difficile* using an enzyme-linked fluorescent immunoassay (Vidas A & B, BioMérieux, Durham, NC, USA) [14] according to the manufacturer's instructions.

Inclusion criteria for cases were as follows: patients with (i) presence of hospital-acquired diarrhea (three or more loose stools per day developing 72 h or more after hospitalization) and positive for* C. difficile* toxin or (ii) negative or indeterminate result for toxins A and B with colonoscopy findings compatible with pseudomembranous colitis (presence of raised yellowish-white 2 to 10 mm plaques overlying erythematous and edematous plaques). Controls were defined as patients with hospital-acquired diarrhea, stools negative for* C. difficile *toxins A and B, and absence of colonoscopic findings characteristic of CDI. Case and control groups were matched in a proportion of 1 : 1 by age (±5 years), sex, and unit of hospitalization (medical or surgical ward). Outpatients, patients aged 15 years or younger, and cases without matched controls were excluded.

Independent variables retrieved from a standardized case report form included types of comorbidity; severity of comorbid conditions according to the Charlson comorbidity index [15]; institutionalization; use of proton pump inhibitors; enteral tube feeding; treatment with cytotoxic chemotherapy in the previous 6 weeks; hospitalization in the previous 6 months; gastrointestinal tract surgery in the previous 30 days; any antibiotic treatment in the previous 30 days; systemic signs and symptoms at the onset of diarrhea (fever, lethargy, dehydration, and tachycardia); distinct gastrointestinal symptoms at the onset of diarrhea (abdominal pain, abdominal distension, anorexia, nausea, and hematochezia); presence of leukocytosis or leukopenia at the onset of diarrhea; serum creatinine, plasma C-reactive protein, plasma albumin, and serum lactate levels and presence of fecal leukocytes (≥1 white blood cell per high-power field) at the onset of diarrhea.

A composite endpoint of all-cause in-hospital mortality, intensive care unit admission, and treatment failure (defined as persistent symptoms of CDI after 5 days of uninterrupted therapy) was used to evaluate factors associated with poor prognosis among patients with documented CDI.

### 2.2. Statistical Analysis

All independent variables with *P* values < 0.10 in the univariate analysis were included in stepwise multiple logistic regression to identify factors associated with* C. difficile* infection among patients with hospital-acquired diarrhea and with poor prognosis among patients with documented* C. difficile* colitis. In the multivariate model, independent variables were eliminated from the highest to the lowest *P* value but remained in the model if their *P* value was <0.05. Odds ratios (ORs) were estimated along with the 95% confidence intervals (CIs). STATA version 12 (StataCorp LP, USA) was used for statistical analysis.

### 2.3. Ethical Issues

The Institutional Research Ethics Committee of Hospital de Clínicas de Porto Alegre approved the study protocol and waived the need for informed consent.

## 3. Results

**Figure 1:**
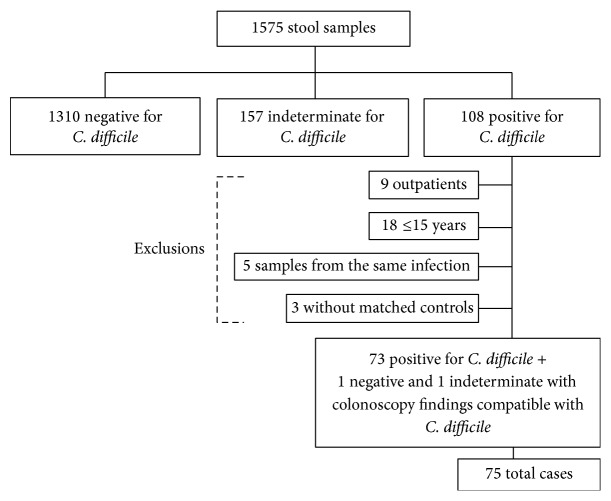
Flow chart showing enrollment of the study patients. ^*∗*^1310 toxin-negative samples belonged to 1244 patients. ^*∗∗*^157 toxin-indeterminate samples belonged to 149 patients. ^*∗∗∗*^108 toxin-positive samples belonged to 103 patients.

During the study period, 1575 stool samples were analyzed. Of these, 108 were positive for toxins A or B, 1310 were negative, and 157 were indeterminate. After applying the exclusion criteria, 75 patients with CDI were identified (Figure 1). These patients were compared with 75 matched controls as described in Section 2. Patient baseline characteristics according to group are summarized in Table 1. With the exception of a higher prevalence of solid organ cancer in the CDI group, there were no significant differences between the two groups in the baseline characteristics. All-cause 30-day mortality was also similar in both groups.

Results of univariate analysis of factors associated with* C. difficile* infection among adult patients with hospital-acquired diarrhea are presented in Table 2. Previous antibiotic treatment (*P* = 0.002), presence of abdominal distension (*P* = 0.006), and presence of fecal leukocytes (*P* = 0.03) occurred significantly more frequently in patients with CDI. HIV infection (*P* = 0.01) and presence of anorexia (*P* = 0.01) were statistically more common in patients with diarrhea of other causes. Multivariate analysis (Table 3) identified previous antibiotic treatment (OR, 13.3; 95% CI, 1.40–126.90), presence of abdominal distension (OR, 3.85; 95% CI, 1.35–10.98), and presence of fecal leukocytes (OR, 8.79; 95% CI, 1.41–54.61) at the onset of diarrhea as predictors of* C. difficile* infection. However, the presence of anorexia was negatively associated with* C. difficile* infection (OR, 0.15; 95% CI, 0.03–0.66). The results of univariate evaluation of factors associated with poor prognosis of CDI are presented in Table 4. Enteral tube feeding (*P* = 0.004) was more frequent in patients with poor prognosis based on the composite endpoint of in-hospital mortality, intensive care unit admission, and treatment failure. Low plasma albumin levels tended to be associated with poor prognosis (*P* = 0.06). However, according to multivariate analysis, enteral tube feeding was the only factor independently associated with poor outcome (OR, 3.75; 95% CI, 1.24–11.29) (Table 5).

## 4. Discussion

The epidemiology of CDI has changed over time and between countries. It is therefore essential to monitor the characteristics of patients at risk of infection. In our multivariate analysis, we have identified three risk factors for CDI in a Brazilian tertiary care hospital, namely, previous antibiotic treatment, presence of fecal leukocytes, and abdominal distension. Curiously, anorexia was negatively associated with CDI, possibly because of a higher prevalence of this finding among patients with hospital-acquired diarrhea of other causes (such as underlying illness, gastrointestinal infections other than CDI, medications, and enteral hyperalimentation) [16].

Previous observational studies with distinct subject groups have reported results convergent with those obtained in the present case-control study. Prior treatment with antimicrobials is considered the main risk factor for CDI: modification of the normal gut flora by antibiotic administration plays an important role in CDI pathogenesis [17]. Additionally, fecal leukocyte testing, which has been proposed as a rapid means of differentiating infective from noninfective diarrhea, might be a useful predictor of* C. difficile* diarrhea in clinical settings where* C. difficile* testing is not available or too time consuming [18]. For example, Fekety and Shah have proposed an algorithm that includes fecal leukocyte testing for diagnosing and managing hospitalized patients with antibiotic-related diarrhea and* C. difficile* colitis [19]. Abdominal distension is frequently associated with fulminant colitis or paralytic ileus in the context of hospital-acquired diarrhea; according to our study, this complication seems to be more frequent in patients with CDI than in those with diarrhea secondary to other causes.

One finding worth highlighting is that, in our study, proton pump inhibitor therapy was not associated with* C. difficile* colitis. In previous studies, proton pump inhibitor therapy has been shown to be a risk factor for* C. difficile* colitis, likely because it decreases the barrier to colonization by vegetative forms of* C. difficile* [20, 21]. However, Henrich et al. found no association between gastric acid suppression and severe* C. difficile* colitis and attributed this to the acid resistance of* C. difficile* spores [22].

In our study, enteral tube feeding was significantly associated with the development of complications of* C. difficile* colitis. Prolonged enteral tube feeding with the use of elemental diets is an additional common, but relatively unrecognized, contributor to the development of* C. difficile* colitis. Although this is usually attributed to the provision by enteral feeding of a high-frequency portal for inoculation of* C. difficile* spores deep into the gut by healthcare workers, it may be simply accounted for by the fact that patients requiring enteral feeding are usually sicker, at higher risk of complications, and more often taking antibiotics than patients not requiring enteral feeding. Nevertheless, some enteral tube feeding diets are totally absorbed within the small intestine and therefore deprive the colonic microbiota of their source of nutrition, namely, dietary fiber, fructose oligosaccharides, and resistant starch. The resultant suppression of colonic fermentation leads to suppression of the “good” bacteria, such as butyrate producers (butyrate being essential for colonic mucosal health) and bifidobacteria and the creation of a “permissive” environment for* C. difficile* colonization and subsequent severe infection [23, 24].

This study has some limitations, mainly related to the single-center observational design. Selection bias is always a possibility in case-control studies, because the selection of controls is often challenging. We used a diagnostic tool with high specificity (98%) and only moderate sensitivity (69.4%) for CDI [14]; consequently, it is probable that our sample failed to include less severe cases of CDI. Nevertheless, our strategy of selecting appropriate controls with non-*C. difficile*-related hospital-acquired diarrhea and matching them to our study subjects was designed to minimize the possibility of selection bias by ensuring that the distributions of possible confounders did not differ substantially between cases and controls [25]. Moreover, measurement of variables and outcomes with previously defined objective criteria and use of standardized data collection for both cases and controls minimized the possibility of systematic errors.

In conclusion, our study has shown that previous antibiotic use and presence of fecal leukocytes in patients with abdominal distension and diarrhea are important predictors of* C. difficile* colitis in the hospital setting. Patients receiving enteral tube support are more likely to develop complications of CDI.

## Figures and Tables

**Table 1:** Baseline characteristics of patients with *Clostridium difficile* colitis and controls with hospital-acquired diarrhea of other causes.

Variable	CDI group (*N* = 75)	Control group (*N* = 75)
Median age, years (range)	54.4 (18.0–91.0)	53.3 (16.0–88.0)
Male	36 (48.0)	36 (48.0)
Unit in which hospitalized		
Medical ward	60 (80.0)	60 (80.0)
Surgical ward	15 (20.0)	15 (20.0)
Relevant comorbidities^1^		
Diabetes mellitus	13 (17.3)	15 (20.0)
Chronic renal disease	10 (13.3)	11 (14.6)
Solid organ cancer	23 (30.6)	8 (10.6)
Hematological cancer	10 (13.3)	10 (13.3)
Solid organ transplant	5 (6.6)	5 (6.6)
Bone narrow transplant	3 (4.0)	5 (6.6)
Deaths^2^	14 (18.6)	13 (17.3)
Median length of hospital stay (days)	50.3	33.4
Median duration of antibiotic use (days)	14.5	15.8
Number of antibiotics used		
None	4 (5.3)	12 (16.0)
One	16 (21.3)	16 (21.3)
Two	12 (16.0)	13 (17.3)
Three	20 (26.6)	12 (16.0)
Four or more	23 (30.6)	22 (29.3)

Data presented as *n* (%) unless otherwise indicated. CDI: *Clostridium difficile* infection. ^1^Most prevalent comorbidities among cases and controls (*n* ≥ 2). ^2^Death from all causes.

**Table 2:** Factors associated with *Clostridium difficile* infection in adult patients with hospital-acquired diarrhea according to univariate logistic regression.

Variable	CDI group (*n* = 75)	Control group (*n* = 75)	OR (95% CI)	*P* value
Charlson comorbidity index, median (IQR)	3.0 (2.0–5.0)	2.0 (1.0–5.0)	1.68 (0.66–4.24)	0.22
HIV infection	5 (6.6)	15 (20.0)	0.28 (0.07–0.89)	0.01
Institutionalization	1 (1.3)	3 (4.0)	0.32 (0.006–4.17)	0.31
Proton pump inhibitor use	53 (70.6)	44 (58.6)	1.69 (0.81–3.54)	0.12
Enteral tube feeding	30 (40.0)	25 (33.3)	1.33 (0.64–2.74)	0.39
Cytotoxic chemotherapy in the previous 6 weeks	19 (25.3)	13 (17.3)	1.61 (0.68–3.90)	0.23
Hospitalization in the previous 6 months	49 (65.3)	48 (64.0)	1.06 (0.51–2.18)	0.86
Gastrointestinal surgery in the previous 30 days	7 (9.3)	5 (6.6)	1.44 (0.37–6.03)	0.54
Antibiotic treatment in the previous 30 days	73 (97.3)	62 (82.6)	7.65 (1.61–71.61)	0.002
Fever	17 (22.6)	12 (16.0)	1.53 (0.62–3.84)	0.30
Lethargy	4 (5.3)	2 (2.6)	2.05 (0.28–23.29)	0.40
Dehydration	9 (12.0)	6 (8.0)	1.56 (0.46–5.65)	0.41
Tachycardia	5 (6.6)	3 (4.0)	1.71 (0.31–11.41)	0.46
Abdominal pain	26 (34.6)	26 (34.6)	1.00 (0.48–2.06)	1.00
Abdominal distension	24 (32.0)	10 (13.3)	3.05 (1.26–7.79)	0.006
Anorexia	6 (8.0)	17 (22.6)	0.29 (0.09−0.86)	0.01
Nausea	7 (9.3)	14 (18.6)	0.44 (0.14–1.28)	0.09
Hematochezia	6 (8.0)	2 (2.6)	3.17 (0.54–32.94)	0.14
Leukocytosis or leukopenia	38 (50.6)	27 (36.4)	1.78 (0.88–3.63)	0.08
Initial serum lactate, mmol/L, median (IQR)	1.1 (0.85–2.2)	0.75 (0.6–0.95)	0.40 (0.59–1.81)	0.10
Initial plasma CPR, mg/L, median (IQR)	70.5 (36.0–157.0)	80.5 (35.0–141.0)	1.001 (0.99–1.006)	0.38
Hypoalbuminemia^*∗*^	44 (58.6)	49 (65.3)	1.01 (0.45–2.27)	0.96
Presence of fecal leukocytes	9 (12.0)	3 (4.0)	4.35 (1.11–17.05)	0.03

Data presented as *n* (%) unless otherwise indicated. CDI: *Clostridium difficile* infection; CPR: C-reactive protein; OR: odds ratio; CI: confidence interval; IQR: interquartile range (P25–P75). ^*∗*^Initial plasma albumin level < 3 g/dL.

**Table 3:** Factors associated with *Clostridium difficile* infection among adult patients with hospital-acquired diarrhea according to multivariate logistic regression.

Variable	OR (95% CI)	*P* value

Antibiotic treatment in the previous 30 days	13.3 (1.40–126.90)	0.01
Anorexia	0.15 (0.03–0.66)	0.01
Abdominal distension	3.85 (1.35–10.98)	0.01
Presence of fecal leukocytes	8.79 (1.41–54.61)	0.02

OR: odds ratio; CI: confidence interval.

Variables entered into the model: HIV infection, antibiotic treatment in the previous 30 days, abdominal distension, anorexia, nausea, leukocytosis or leukopenia, and presence of fecal leukocytes.

**Table 4:** Factors associated with poor prognosis^*∗*^ among 75 hospitalized adult patients with *Clostridium difficile* colitis according to univariate logistic regression.

Variable	Poor prognosis group (*n* = 32)	Good prognosis group (*n* = 43)	OR (95% CI)	*P* value
Age, years, median (IQR)	59.0 (39.5–76.0)	55.0 (35.0–67.0)	1.008 (0.98–1.03)	0.46
Charlson comorbidity index, median (IQR)	3.0 (2.0–5.0)	2.0 (1.0–5.0)	1.68 (0.66–4.24)	0.22
HIV infection	3 (9.3)	2 (4.6)	2.12 (0.33–13.50)	0.42
Proton pump inhibitors use	25 (78.1)	28 (65.1)	1.91 (0.67–5.44)	0.22
Enteral tube feeding	19 (59.3)	11 (25.5)	4.25 (1.59–11.36)	0.004
Cytotoxic chemotherapy in the previous 6 weeks	6 (18.7)	13 (30.2)	0.53 (0.17–1.60)	0.26
Hospitalization in the previous 6 months	19 (59.3)	30 (69.7)	0.63 (0.24–1.65)	0.35
Gastrointestinal surgery in the previous 30 days	5 (15.6)	2 (4.6)	3.79 (0.68–20.99)	0.12
Length of antibiotic treatment, days, median (IQR)	10.0 (7.0–15.0)	10.0 (7.0–14.0)	1.07 (0.96–1.19)	0.19
Fever	9 (28.1)	8 (18.6)	1.71 (0.57–5.08)	0.33
Lethargy	2 (6.2)	2 (4.6)	1.36 (0.18–10.25)	0.76
Dehydration	4 (12.5)	5 (11.6)	1.08 (0.26–4.41)	0.90
Tachycardia	3 (9.3)	2 (4.6)	2.12 (0.33–13.50)	0.42
Abdominal pain	8 (25.0)	18 (41.8)	0.46 (0.16–1.26)	0.13
Abdominal distension	13 (40.6)	11 (25.5)	1.99 (0.74–5.32)	0.17
Anorexia	2 (6.2)	4 (9.3)	0.65 (0.11–3.78)	0.63
Nausea	2 (6.2)	5 (11.6)	0.50 (0.09–2.79)	0.43
Hematochezia	1 (3.1)	5 (11.6)	0.24 (0.02–2.20)	0.21
Leukocytosis or leukopenia	13 (40.6)	25 (58.1)	0.49 (0.19–1.24)	0.13
Initial serum creatinine, mg/dL, median (IQR)	1.1 (0.7–3.05)	0.8 (0.6–1.3)	1.27 (0.93–1.72)	0.12
Initial plasma CPR, mg/L, median (IQR)	63.0 (33.5–174.0)	84.5 (47.0–157.0)	0.99 (0.99–1.00)	0.75
Initial plasma albumin, g/dL, median (IQR)	2.7 (2.3–3.3)	3.2 (2.7–3.6)	0.46 (0.21–1.03)	0.06
Initial serum lactate, mmol/L, median (IQR)	1.1 (0.85–2.2)	0.75 (0.6–0.95)	0.40 (0.59–1.81)	0.10

Data presented as *n* (%) unless otherwise indicated. CPR: C-reactive protein; OR: odds ratio; CI: confidence interval; IQR: interquartile range (P25–P75).

^*∗*^Composite endpoint that includes in-hospital mortality, ICU admission, and treatment failure.

**Table 5:** Factors associated with poor prognosis^*∗*^ among 75 hospitalized adult patients with *Clostridium difficile* colitis according to multivariate logistic regression.

Variable	OR (95% CI)	*P* value
Enteral tube feeding	3.75 (1.24–11.29)	0.01

Variables entered into the model: enteral tube feeding and initial plasma albumin concentration. OR: odds ratio; 95% CI: 95% confidence interval.

^*∗*^Composite endpoint that includes in-hospital mortality, ICU admission, and treatment failure.
